# Characterization of Experimental Oro-Nasal Inoculation of Seba’s Short-Tailed Bats (*Carollia perspicillata*) with Bat Influenza A Virus H18N11

**DOI:** 10.3390/v12020232

**Published:** 2020-02-19

**Authors:** Marco Gorka, Jan Schinköthe, Reiner Ulrich, Kevin Ciminski, Martin Schwemmle, Martin Beer, Donata Hoffmann

**Affiliations:** 1 Institute of Diagnostic Virology, Friedrich-Loeffler-Institut, 17493 Greifswald, Insel Riems, Germany; marco.gorka@fli.de; 2 Department of Experimental Animal Facilities and Biorisk Management, Friedrich-Loeffler-Institut, 17493 Greifswald, Insel Riems, Germany; Jan.Schinkoethe@vetmed.uni-leipzig.de (J.S.); reiner.ulrich@vetmed.uni-leipzig.de (R.U.); 3 Institute of Veterinary Pathology, Faculty of Veterinary Medicine, Leipzig University, 04109 Leipzig, Germany; 4 Institute of Virology, Medical Center, University of Freiburg, 79104 Freiburg, Germany; kevin.ciminski@uniklinik-freiburg.de (K.C.); martin.schwemmle@uniklinik-freiburg.de (M.S.); 5 Faculty of Medicine, University of Freiburg, 79104 Freiburg, Germany

**Keywords:** bats, virus, bat Influenza A viruses, host species, experimental infection, pathogenesis, transmission

## Abstract

In 2012 and 2013, the genomic sequences of two novel influenza A virus (IAV) subtypes, designated H17N10 and H18N11, were identified via next-generation sequencing in the feces of the little yellow-shouldered fruit bat (*Sturnira lilium*) and the flat-faced fruit-eating bat (*Artibeus planirostris*), respectively. The pathogenesis caused by these viruses in their respective host species is currently insufficiently understood, which is primarily due to the inability to obtain and keep these bat species under appropriate environmental and biosafety conditions. Seba’s short-tailed bats (*Carollia perspicillata*), in contrast, are close relatives and a natural H18N11 reservoir species, with the advantage of established animal husbandry conditions in academic research. To study viral pathogenesis in more detail, we here oro-nasally inoculated Seba’s short-tailed bats with the bat IAV H18N11 subtype. Following inoculation, bats appeared clinically healthy, but the histologic examination of tissues revealed a mild necrotizing rhinitis. Consistently, IAV-matrix protein and H18-RNA positive cells were seen in lesioned respiratory and olfactory nasal epithelia, as well as in intestinal tissues. A RT-qPCR analysis confirmed viral replication in the conchae and intestines as well as the presence of viral RNA in the excreted feces, without horizontal transmission to naïve contact animals. Moreover, all inoculated animals seroconverted with low titers of neutralizing antibodies.

## 1. Introduction

Influenza A viruses (IAVs) originate from aquatic waterfowl and are important animal pathogens and zoonotic agents, circulating in a broad range of avian and mammalian species [1]. Although spill-over infections from animals to humans are possible, these events are exceedingly rare [2,3,4,5]. Bats (order *Chiroptera*) are well known as an important reservoir for various zoonotic pathogens like lyssa-, henipa- or filoviruses; however, until recently they were not considered to harbor IAVs [6,7]. This view changed in 2012 and 2013, when two novel IAVs genomes were identified by next-generation sequencing in the feces of the little yellow-shouldered fruit bat (*Sturnira lilium*) from Guatemala and the flat-faced fruit-eating bat (*Artibeus planirostris*) from Peru, subsequently designated as H17N10 and H18N11 [8,9]. These bat-derived IAVs exhibited unprecedented characteristics, since both subtypes express hemagglutinin (HA) and neuraminidase (NA) proteins unable to bind sialic acids residues [10,11,12,13,14,15]. Instead, recent research by Karakus et al. showed that the bat influenza HA proteins H17 and H18 mediate cell entry by utilizing major-histocompatibility-complex class II (MHC-II) molecules [16]. In contrast, the function of the bat IAV NA proteins N10 and N11 remain elusive [11,13,17]. Furthermore, the tropism of H18N11 and the pathogenesis in its natural reservoir is insufficiently understood, as there is currently only one study available focusing on the Neotropical Jamaican fruit bat (*Artibeus jamaicensis*). In this study, viral replication was found to occur in the gastrointestinal tract of infected Jamaican fruit bats, followed by the shedding of wildtype H18N11 via their feces [18]. Intriguingly, seroepidemiological screenings revealed a prevalence of H18-specific antibodies in various Central and South American bat species, including Seba’s short-tailed bats (*Carollia perspicillata*) [9]. However, as viral RNA could not be isolated from any Seba’s short-tailed bat sample, the relevance of this bat species within the H18N11 ecology remains unknown. We here studied the H18N11-induced pathogenesis in Seba’s short-tailed bats, a well established bat infection model [19,20,21].

## 2. Materials and Methods 

### 2.1. Ethics Statement

The animal experiments described here were approved by the State Office for Agriculture, Food Safety, and Fishery of Mecklenburg-Western Pomerania under registration number LVL MV TSD/ 7221.3-1-021/18.

### 2.2. Virus 

Animals were inoculated using recombinant H18N11 virus generated as described [18]. In brief, the eight plasmid pHW2000-based rescue system was used to generate infectious A/flat-faced bat/Peru/033/2010 (H18N11) which could be further passaged on cell culture. In detail, the recombinant bat influenza A virus H18N11 was generated by transfecting HEK293T cells (American Type Culture Collection ATCC, Manassas, VA, USA) seeded in 6-well plates with the eight pHW2000-based rescue plasmids (300 ng of each plasmid per 6-well). Forty-eight hours post transfection, the cell supernatant was collected and concentrated by ultracentrifugation through a sucrose gradient (25000 rpm, 2 h, 8 °C). Stocks were generated by infecting canine RIE1495 cells (canine epithelial cell line, stored in the “Collection of Cell Lines in Veterinary Medicine” (CCLV) at the Friedrich-Loeffler Institute in Greifswald-Insel Riems, Germany with the code CCLV-RIE 1495) at an MOI of 0.1 with concentrated HEK293T rescue supernatant for 48 h. The viral titers were determined by immunofluorescence assay, using H18-specific antibodies.

### 2.3. Bat Experiment and Sampling

**Figure 1:**
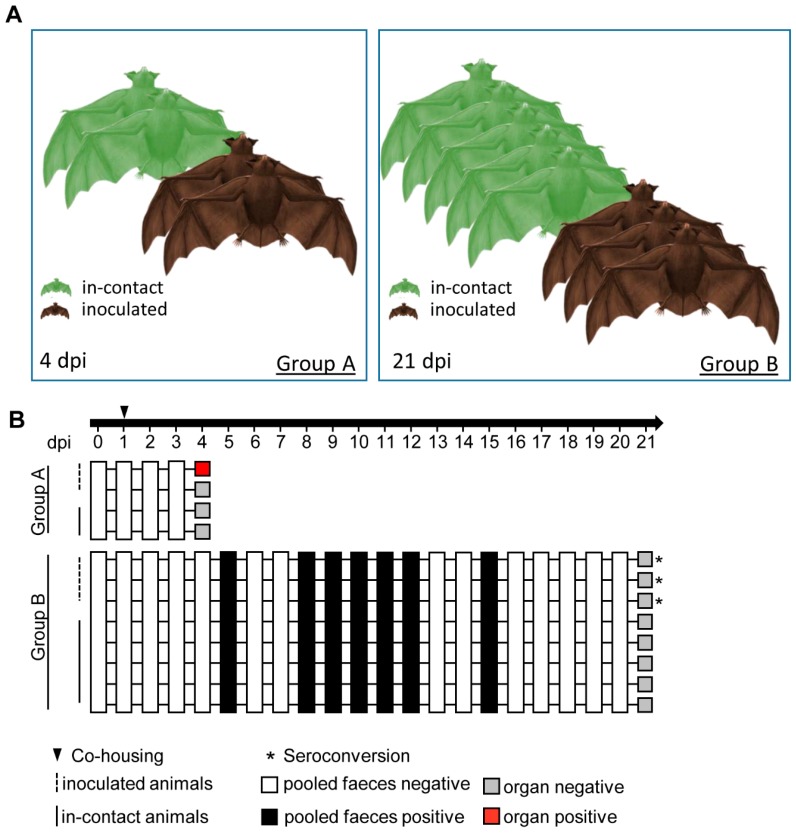
Experimental design. (**A**) Group A consisted of two inoculated bats in direct contact to naïve bats co-housed in one cage. At 4 dpi, the animals were euthanized and the organ material analyzed. Group B involved three inoculated bats co-housed with five naïve animals until 21 dpi. Group C (not presented) included two Seba’s short-tailed bats for negative tissue control. (**B**) Index bats were inoculated with 10^5.5^ TCID_50_ of H18N11 oronasally. Pooled feces samples were taken at the indicated time points. White squares indicate absence, black squares the presence of viral RNA in feces samples. Grey squares show absence, the red squares presence of H18N11 RNA in at least one organ. Asterisks indicate seroconversion.

All experiments were conducted following internal standard guidelines under biosafety level 3 conditions at the Friedrich-Loeffler-Institut. Fourteen healthy and influenza-antibody-negative (see serology section below) Seba’s short-tailed bats (*Carollia perspicillata*) were enrolled in this study (Figure 1A). These individuals were divided into group A (*n =* 4), B (*n =* 8) and C (*n =* 2). Inoculation of inhalatively anesthetized (5% isoflurane) bats was performed by the oro-nasal route with 10^5.5^ tissue culture infectious dose (TCID_50_) of H18N11 in 50 µl in two individuals of group A, and three individuals in group B. The sentinel bats of both groups were housed together with the inoculated ones 24 h after infection, in order to assess virus spread. Clinical signs (nasal discharge, reduced activity, neurological symptoms, dyspnoe), room temperature and relative humidity were monitored daily, as well as collection of pooled fecal samples. All animals in group A were sacrificed at 4 dpi and animals in group B were sacrificed at 21 dpi, followed by necropsy. Samples from the conchae, trachea, lung (left caudal lung lobe, right cranial lung lobe), heart, kidney, liver, intestine, olfactory bulb, cerebrum, cerebellum and thigh muscles were collected and stored at −80 °C until further processing for virological assays. Blood samples for serology were collected during the terminal bleeding procedure. 

### 2.4. Organ Homogenization

To start with, 2 mL collection tubes were filled with a stainless steel bead (diameter 5 mm, TIS Wälzkörpertechnologie GmbH, Gauting, Germany) and 1 mL of DMEM supplemented with 10% fetal bovine serum (FBS) and antibiotics (1% penicillin-streptomycin [PenStrep], Biochrom GmbH, Berlin Germany). Homogenization was performed via TissueLyser II (Qiagen, Hilden, Germany) for 2 min. The supernatants, for RNA extractions, were acquired by centrifuging at 13,000 rpm for 2 min.

### 2.5. RNA Isolation

Organ Samples: Viral RNA extraction was achieved through solubilization of 250 μL of the supernatant of organ homogenates with 750 µl TRIzol LS Reagent (Life Technologies, Carlsbad, CA, USA). After the addition of 200 µL ROTIPURAN (Carl Roth, Karlsruhe, Germany), phase separation was attained. The following steps were completed with the NucleoMag Vet kit (Macherey-Nagel, Düren, Germany) according to the manufacturer’s instructions on a Biosprint 96 platform (Qiagen). 

Fecal samples: Viral RNA extraction of pooled fecal samples (group A pool and goup B pool) was achieved with the MasterPure™ Complete DNA and RNA Purification Kit (Lucigen, Middleton, WI, USA) according to the manufacturer’s instructions, after a dilution of the samples by the factor 1:1000 in PBS.

### 2.6. RT-qPCR 

The real-time RT-PCRs (RT-qPCR) of all organ and fecal samples were performed as described before [22]. In brief, a generic PB1 assay was used to determine the quantification cycle (Cq) using the one-step RT-qPCR Kit qScript™ XLT One-Step RT-qPCR ToughMix^®^ (Quantabio, Beverly, MA USA). The RT-qPCR assay was optimized for using a total volume of 12.5 µl. The reaction was run on a bio-rad cycler Cfx96 machine (Bio-Rad Laboratories, Inc. Hercules, CA, USA). Individual amplification controls on the basis of artificial spiked RNA (fecal samples, [23]) or beta actin (organ samples modified [24]) were used to evaluate inhibitory effects.

### 2.7. Virus Isolation 

Virus isolation attempts were performed using RIE1495 cells and homogenized organ samples, which scored positive for viral RNA. Briefly, 50µL supernatant from the homogenized organ was applied onto 12.5 cm^2^ cell culture flask (Corning, Corning, NY, USA). Afterwards four blind passages of potential infected cells were done, followed by a RT-qPCR based analysis.

### 2.8. Serology

Serum samples from all animals were heat inactivated at 56°C for 30 min and analyzed using an indirect immunofluorescence test and a virus neutralization assay. After fixation of RIE1495 cells and RIE1495 cells infected with A/flat-faced bat/Peru/033/2010 (H18N11) using aceton methanol (1:1 vol%), the cells were incubated for one hour with the bat sera. After three washing steps using PBS, goat anti-bat IgG (H+L) secondary antibody (Novus Biologicals, Littleton, CO, USA) was applied for one hour at room temperature. After an additional three washing steps with PBS, chicken anti-goat Alexa 488 (ThermoFisher scientific, Waltham, MA, USA) was added and incubated for one hour at room temperature. 

Briefly, the neutralization assay was performed with 50 µl of medium containing VSV*ΔG-H18 [25] at a concentration of 10^3.3^ TCID_50_ that was mixed with the same volume of diluted serum. Each serum was prepared in triplicate in a 96-well plate. After incubation of 2 h at 37 °C the dilution was transferred on 100 µL medium and 24 h grown in RIE 1495 cells. The viral replication was assessed after an incubation of 5 days (37 °C, 5% CO_2_) via visualization of GFP expression. Validation was achieved by titration of the virus dilutions. 

### 2.9. Necropsy and Histologic Examination

A complete necropsy with macroscopic evaluation of tissues was done for all animals of this study. Histopathologic, immunohistologic and in situ hybridization workup was performed for all animals necropsied at 4 dpi (group A) and two non-inocluated bats (group C). A specimen of parenchymal organs and the skull were fixed in 4% neutral buffered formaldehyde. The skulls were decalcified (Formical 2000, Quartett Immundiagnostika und Biotechnologie Vertriebs GmbH, Berlin, Germany) and the following organs were processed to formalin fixed, paraffin embedded (FFPE) tissue blocks: four standardized coronal nasal sections with minimal adjustment to size ratios of *Carollia perspicillata* skulls as described elsewhere [16,18], the middle and inner ear, parotis, eye, oral cavity, esophagus, trachea, thyroidea, left caudal lung lobe, right cranial lung lobe, heart, liver, pancreas, stomach, small intestine with jejunal Peyer’s patches, mesenteric lymph nodes, kidney, adrenal glands, bulbus olfactorius, cerebrum, cerebellum and bone marrow. Two to four micron-thick sections were cut and stained with hematoxylin and eosin. All specimens were evaluated for histopathologic lesions using an Axio Imager M2 microscope equipped with 10×, 20×, and 40× Plan Neofluar objectives and an AxioCam ICc3 3.3-megapixel digital camera (Carl Zeiss Microscopy GmbH, Jena, Germany).

### 2.10. Immunohistochemistry 

To visualize IAV-matrix protein, immunohistochemistry (IHC) was performed using the avidin-biotin-peroxidase-complex (ABC) method (Vectastain^®^ Elite ABC Kit Standard, Vector Laboratories, Burlingame, CA, USA) with citric buffer (10 mM, pH 6,0) pre-treatment, a monoclonal mouse anti-IAV-matrix protein immunoglobulin G1 containing hybridoma supernatant (dilution 1:10, clone M2-1C6-4R3 [26]), with 3-amino-9-ethyl-carbazol as chromogen and hematoxylin counterstain. Archival formalin-fixed and paraffin embedded (FFPE) tissues of one H18N11-infected Jamaican fruit bat [18], as well as H18N11-infected and mock-inoculated RIE1495 cell pellets served as positive and negative controls, respectively. Furthermore, the primary antibody was replaced by Tris-buffered saline on a following section as a second negative control for each evaluated tissue section.

### 2.11. In Situ Hybridization

In situ hybridization (ISH) was performed for the detection of IAV (A/flat-faced bat/Peru/033/2010 (H18N11)) H18-specific RNA using a RNAscope^®^ 2.5 assay (ACDbio, Newark, CA, USA) with target retrieval and protease pretreatment, RNAscope^®^ 2.5 HD Reagent Kit—RED, the HybEZ™ hybridization system with a 20ZZ probe named V-Bat-Influenza-HA targeting base pairs 26-1132 of gene bank accession number CY125945.1 and Fast Red as chromogen and hematoxylin counterstain, as described previously [16,18]. 

## 3. Results

A recent study reported that H18N11 replicates especially in the lamina propria of the small intestine and the follicle-associated epithelium of the jejunal Peyer’s patches of infected Jamaican fruit bats [18]. Although these acutely infected animals shed high viral loads via the rectal route, no inflammatory lesions were observed. To determine tissue tropism and pathogenesis in the related Seba’s short-tailed bats (*Carollia perspicillata*) at different time points after infection, animals were split into three groups: Group A consisted of four bats sacrificed at 4 dpi, from which two were initially inoculated and two others that remained naïve in order to monitor viral transmission (Figure 1A). Group B comprised three inoculated bats and five co-housed naïve contact animals that were sacrificed at 21 dpi. Two naïve bats were kept as controls in group C. 

Following oro-nasal inoculation of the group A bats, none of the inoculated index or naïve contact animals exhibited clinical signs of disease and viral RNA was not present in the feces (Figure 1B). One of two H18N11-inoculated group A individuals was found viral RNA-positive in the nasal conchae (quantification cycle value (Cq) 33.62) and in the colon (Cq 34.72) (Table 1), whereas all other tested organ samples were PCR-negative. However, despite detection of viral RNA in some tissue samples, all attempts to isolate the infectious virus from these organs failed.

**Figure 2:**
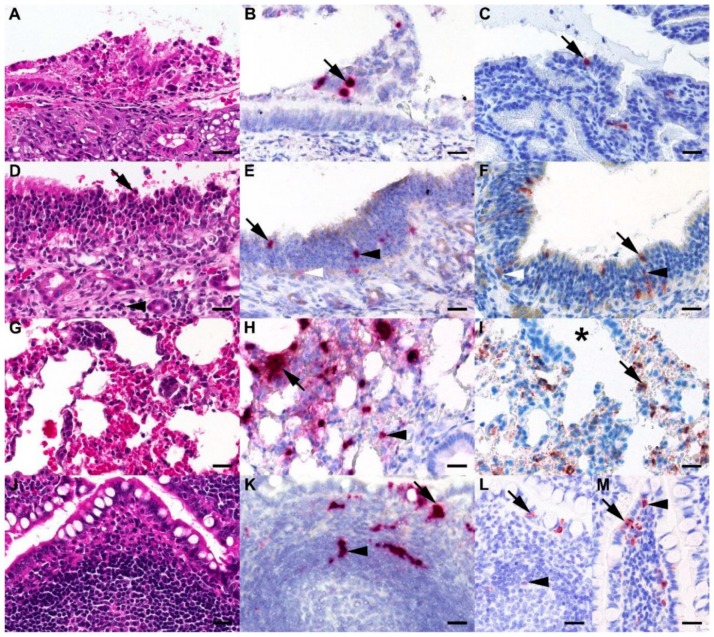
Histologic lesions exhibit H18-specific RNA signals and IAV-matrix protein immunoreactivity in the upper and lower respiratory tract of H18N11-infected Seba’s short-tailed bat (*Carollia perspicillata*) at 4 dpi. (**A**) Bat, rostral nose. Mild, focal, acute, necrotizing rhinitis with erosion of the respiratory epithelium and sloughing of cells. (**B**) Bat, rostral nose. Oligofocal single H18-specific RNA signals in luminal debris (arrow). (**C**) Bat, rostral nose. Oligofocal strong intracytoplasmic and intranuclear IAV-matrix protein immunoreactive respiratory epithelial cells (arrow). (**D**) Bat, caudal nose. Mild, focal, acute, necrotizing rhinitis with pyknotic cells (arrow) and mild multifocal infiltration of neutrophils (arrowhead) in the Bowman’s glands rich lamina propria. (**E**,**F**) Bat, caudal nose. Oligofocal H18-specific RNA signals and strong intracytoplasmic and intranuclear IAV-matrix protein immunoreactive cells interpreted as sustentecular cells (black arrow), olfactory receptor neurons (black arrowhead) and basal cells (white arrowhead). (**G**) Bat, right cranial lung lobe. Normal appearing lung tissue with alveolar spaces. (**H**) Bat, right cranial lung lobe. Multifocal H18-specific RNA signals in intra-alveolar cellular clusters (arrow) and in single cells (arrowhead). (**I**) Multifocal strong IAV-matrix protein reactive cells (arrow) in areas associated with respiratory bronchioles (asterisk). (**J**) Bat, jejunal Peyer’s patch (JPP). Follicle-associated epithelium (FAE) and a lymphocyte-rich subepithelial dome region of a normal appearing JPP is depicted. (**K**) Bat, JPP. Multifocal H18-specific RNA signals are seen in FAE (arrow) and in the subepithelial dome regions (arrowhead). (**L**) Bat, JPP. Oligofocal strong intracytoplasmic IAV-matrix protein immunoreactive cells are seen in the FAE (arrow) and faintly intracytoplasmic reactive cells next to a germinal center (arrowhead). (**M**) Bat, jujunal villus. Oligofocal strong intracytoplasmic and intranuclear IAV-matrix protein immunoreactive enterocytes (arrow) and lamina propria associated round cells interpreted as macrophages or dendritic cells (arrowhead) were evident. A, D, G, J, Hematoxylin eosin stain; B, E, H, K, in situ hybridization, target retrival and protease pretreatment, RNAscope^®^ 2.5 assay H18-specific RNA, Fast Red chromogen (red), hematoxylin counterstain (blue), C, F, I, L, M IAV-matrix protein immunohistochemistry, avidin-biotin-peroxidase complex method with 3-amino-9-ethyl-carbazol as chromogen and hematoxylin counterstain; bars, A–M = 20 µm.

No gross lesions were observed in infected and naïve contact bats at 4 dpi, however, histopathologic examination revealed a mild, oligofocal, acute, necrotizing rhinitis in the rostral and caudal coronal sections of the nasal cavity, characterized by erosive alterations of the respiratory epithelium with pyknotic and karyorrhectic cells in the PCR-positive index bat (Figure 2A). Correspondingly, oligofocal H18-specific RNA in luminal debris (Figure 2B) and oligofocal strong IAV-matrix protein positive cells were seen (Figure 2C, Table 1). Erosive alterations were also present in the olfactory epithelium with mild infiltration of neutrophils in the lamina propria (Figure 2D). Oligofocal H18-specific RNA (Figure 2E) and oligofocal strong IAV-matrix protein positive reactions (Figure 2F) were mainly seen in cells interpreted as sustentacular cells. In the lower respiratory tract, multifocal H18-specific RNA signals and IAV-matrix protein immunoreactive cells were seen in clusters and in single cells mostly confined to alveoli closely associated with respiratory bronchioles interpreted as alveolar macrophages and/or pneumocytes type 2 (Figure 2I), despite a lack of unequivocal histopathologic lesions (Figure 2G). Small intestinal villi and jejunal Peyer’s patches (JPP, Figure 2J) showed no alterations. However, multifocal H18-specific RNA signals predominantly in follicle-associated epithelium (FAE) and subepithelial dome regions (Figure 2K), and oligofocal in enteroyctes of small intestinal villi were seen in both infected bats (Table 1). A similar distribution of strong IAV-matrix protein immunoreactive cells was seen in the FAE (Figure 2L) of JPPs and oligofocal within enterocytes and round cells in the lamina propria, interpreted as macrophages or dendritic cells (Figure 2M). No comparable lesions, no H18-specific RNA and no IAV matrix proteins were detectable in the organs of either naïve contact animals.

**Figure 3:**
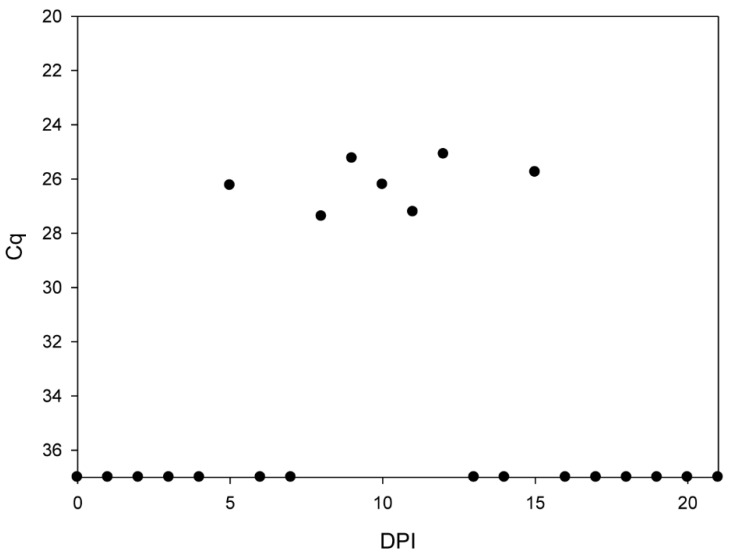
Quantification of IAV RNA loads by RT-qPCR in pooled fecal samples. The amounts of viral genome load were measured by RT-qPCR at the indicated time points. Prior to extraction the guano samples were diluted 1:1000. Calculated values are presented.

While the bats of group A appeared clinically healthy and shed no infectious virus until 4 dpi, two bats of group B developed a green-colored diarrhea between 9 and 21 dpi. Although we could not obtain individual rectal swabs of healthy and diseased bats without compromising their health status due to the stressful catching procedure, a RT-qPCR analysis of pooled fecal samples revealed the presence of viral RNA at 5, between 8 and 12 and 15 dpi (Figure 1B and Figure 3). 

At necropsy, an untypical green to grey-colored and softened ingesta was seen in the colon and rectum in two of the eight bats from group B. Consistent with the observation that viral RNA was shed in the feces only until 15 dpi, the examined tissues of inoculated index and contact bats were found RT-qPCR-negative at 21 dpi. Furthermore, a histopathological analysis and immunohistochemistry of all tissues collected from animals with diarrhea (*n =* 2) found lesion-free, IAV-matrix protein antigen negative reactions. Importantly, aside from the green-colored softened feces, no further symptoms of disease were monitored throughout the course of the experiment. 

Serum samples were taken from all group B bats after euthanasia and tested for the presence of neutralizing antibodies. The sera obtained from all three inoculated index bats revealed a low-titer seroconversion with a 50% neutralization dose (ND50) of 1:20, 1:253 and 1:16, whereas all naïve contact bats were tested seronegative (Figure 1B).

## 4. Discussion

Here, we show that the Seba’s short-tailed bat—a species showing positive seroreactivities against H18N11 in nature [9]—is susceptible to experimental oro-nasal infection with H18N11. The detection of viral RNA in tissues of the upper respiratory tract and the intestines in one of the inoculated bats at 4 dpi might indicate a productive oro-nasal and intestinal infection, although we cannot entirely exclude the possibility of detecting residual inoculum in these animals. Upon histopathological analysis, the PCR-positive bats exhibited mild necrotizing alterations confined to respiratory and olfactory epithelium. Moreover, H18-specific RNA signals and IAV-matrix protein immunoreactive cells were present in the nose and gut-associated-lymphoid-tissue (GALT) at early time points (4 dpi), and viral RNA was detected in fecal samples at later time points, which altogether corresponds to the previously suggested gastro-intestinal tissue tropism of H18N11 in Jamaican fruit bats and its spread by the rectal route [18]. Our findings further support the idea of viral replication in MHC-II-positive antigen-presenting cells, such as macrophages and dendritic cells [16,18], which are present in high frequencies at the FAE of Peyer’s patches and other inductive sites of the mucosal immunity [27]. 

During experimentation, some bats—presumably the index animals—developed a mild exsiccosis and catarrhal enteritis, characterized by green-colored diarrhea, and two bats additionally exhibited abnormal softened ingesta at the time of necropsy at 21 dpi. It is open to discussion whether the mild enteritis is a sequela of the IAV infection or a result of animal handling or simply an independent background lesion. 

In contrast to the previous H18N11 infection study performed in Jamaican fruit bats [18], no horizontal transmission occurred among Seba’s short-tailed bats. A generally unlikely, yet possible explanation for this finding could be that the contact bats were exposed to bat IAV before, but their antibody titers dropped below the detection threshold. Continuous re-exposure could have triggered a rapidly spiking and thereafter declining antibody response sufficient to prevent an infection. Such rapidly waning antibody responses after an infection—resulting in seronegativity—have been described before for Marburg virus infections in Egyptian fruit bats [28]. Another possibility could be the advanced age of the bats used in this study. Older bats mount a more efficient antiviral immune response which makes the transmission of viruses between individuals a more challenging task [29]. In this context, the previously described constitutively “always on” type I IFN activity in bat cells [30] might also interfere with viral replication and could hence restrict virus transmission among a group of bats, compared to other mammalian species. Alternatively, the lack of transmission could also be attributed to an *Artibeus* species specificity of H18N11. Although various South American fruit bat species have been found seropositive for H17 and H18 [9], until now full H18N11 genomic sequences or infectious viruses have been only isolated from *Artibeus planirostris* and *Artibeus lituratus* or *Artibeus jamaicensis*, respectively [9,18,31]. Considering this, it is tempting to speculate that species-specific differences in the immunity or the adaptation of H18 to MHC-II molecules of *Artibeus* spp. have influenced the infection outcome. 

In summary, Seba’s short-tailed bats are susceptible to, and develop, mild upper respiratory tract lesions after experimental infection with the bat-derived H18N11 subtype, but transmission to contact bats was not evident. Therefore, Seba’s short-tailed bats most likely do not contribute to transmission cycles of H18N11 in nature.

## Figures and Tables

**Table 1:** Distribution of viral RNA and matrix protein antigen signals in H18N11-infected bats at 4 dpi.

Animal No.	1			2		
Method	PCR	ISH	IHC	PCR	ISH	IHC
Conchae	**33.6**	**1 ^#^**	**1**	-	0	0
Trachea	-	0	0	-	0	0
Lung	-	2	2	-	0	0
Heart	-	0	0	-	0	0
Kidney	-	0	0	-	0	0
Liver	-	0	0	-	0	0
Intestine	**34.7 ^†^**	2 ^*^	2 ^*^	-	2 ^*^	1 ^*^
Olfactory bulb	-	0	0	-	0	0
Cerebrum	-	0	0	-	0	0
Cerebellum	-	0	0	-	0	0
Muscle	-	nd	nd	-	nd	nd

PCR, quantitative real-time polymerase chain reaction; ISH, in situ hybridization; IHC, immunohistochemistry; -, no Cq; nd, not done; ^#^ semiquantitative scoring (1 = focal/oligofocal; 2 = multifocal; 3 = coalescing/diffuse); ^*^ in jejunal Peyer’s patches, score 1 in jejunum, ^†^ in colon.

## References

[1] Webster R.G., Bean W.J., Gorman O.T., Chambers T.M., Kawaoka Y. (1992). Evolution and ecology of influenza A viruses. Microbiol. Rev..

[2] Garten R.J., Davis C.T., Russell C.A., Shu B., Lindstrom S., Balish A., Sessions W.M., Xu X., Skepner E., Deyde V. (2009). Antigenic and Genetic Characteristics of Swine-Origin 2009 A(H1N1) Influenza Viruses Circulating in Humans. Science.

[3] Imai M., Watanabe T., Hatta M., Das S.C., Ozawa M., Shinya K., Zhong G., Hanson A., Katsura H., Watanabe S. (2012). Experimental adaptation of an influenza H5 HA confers respiratory droplet transmission to a reassortant H5 HA/H1N1 virus in ferrets. Nature.

[4] Desselberger U., Nakajima K., Alfino P., Pedersen F.S., Haseltine W.A., Hannoun C., Palese P. (1978). Biochemical evidence that “new” influenza virus strains in nature may arise by recombination (reassortment). Proc. Natl. Acad. Sci. USA.

[5] Herfst S., Schrauwen E.J.A., Linster M., Chutinimitkul S., De Wit E., Munster V.J., Sorrell E.M., Bestebroer T.M., Burke D., Smith D.J. (2012). Airborne Transmission of Influenza A/H5N1 Virus Between Ferrets. Science.

[6] Calisher C.H., Childs J.E., Field H.E., Holmes K.V., Schountz T. (2006). Bats: Important Reservoir Hosts of Emerging Viruses. Clin. Microbiol. Rev..

[7] Wynne J.W., Wang L.F. (2013). Bats and viruses: Friend or foe?. PLoS Pathog..

[8] Tong S., Li Y., Rivailler P., Conrardy C., Castillo D.A.A., Chen L.-M., Recuenco-Cabrera S., Ellison J.A., Davis C.T., York I. (2012). A distinct lineage of influenza A virus from bats. Proc. Natl. Acad. Sci. USA.

[9] Tong S., Zhu X., Li Y., Shi M., Zhang J., Bourgeois M., Yang H., Chen X., Recuenco-Cabrera S., Gómez J. (2013). New World Bats Harbor Diverse Influenza A Viruses. PLOS Pathog..

[10] Sun X., Shi Y., Lu X., He J., Gao G.F., Yan J., Qi J., Gao G.F. (2013). Bat-Derived Influenza Hemagglutinin H17 Does Not Bind Canonical Avian or Human Receptors and Most Likely Uses a Unique Entry Mechanism. Cell Rep..

[11] Li Q., Sun X., Li Z., Liu Y., Vavricka C.J., Qi J., Gao G.F. (2012). Structural and functional characterization of neuraminidase-like molecule n10 derived from bat influenza a virus. Proc. Natl. Acad. Sci. USA.

[12] Zhu X., Yu W., McBride R., Li Y., Chen L.-M., Donis R.O., Tong S., Paulson J.C., Wilson I.A. (2013). Hemagglutinin homologue from H17N10 bat influenza virus exhibits divergent receptor-binding and pH-dependent fusion activities. Proc. Natl. Acad. Sci. USA.

[13] Zhu X., Yang H., Guo Z., Yu W., Carney P.J., Li Y., Chen L.-M., Paulson J.C., Donis R.O., Tong S. (2012). Crystal structures of two subtype N10 neuraminidase-like proteins from bat influenza A viruses reveal a diverged putative active site. Proc. Natl. Acad. Sci. USA.

[14] Gambaryan A., Tuzikov A., Piskarev V., Yamnikova S.S., Lvov D.K., Robertson J., Bovin N.V., Matrosovich M. (1997). Specification of Receptor-Binding Phenotypes of Influenza Virus Isolates from Different Hosts Using Synthetic Sialylglycopolymers: Non-Egg-Adapted Human H1 and H3 Influenza A and Influenza B Viruses Share a Common High Binding Affinity for 6?-Sialyl(N-acetyllactosamine). Virology.

[15] Sauter N.K., Bednarski M.D., Wurzburg B.A., Hanson J.E., Whitesides G.M., Skehel J.J., Wiley N.C. (1989). Hemagglutinins from two influenza virus variants bind to sialic acid derivatives with millimolar dissociation constants: A 500-MHz proton nuclear magnetic resonance study. Biochemistry.

[16] Karakus U., Thamamongood T., Ciminski K., Ran W., Günther S.C., Pohl M., Eletto D., Jeney C., Hoffmann D., Reiche S. (2019). MHC class II proteins mediate cross-species entry of bat influenza viruses. Nature.

[17] Juozapaitis M., Moreira É.A., Mena I., Giese S., Riegger D., Pohlmann A., Höper D., Zimmer G., Beer M., García-Sastre A. (2014). An infectious bat-derived chimeric influenza virus harbouring the entry machinery of an influenza A virus. Nat. Commun..

[18] Ciminski K., Ran W., Gorka M., Lee J., Malmlov A., Schinköthe J., Eckley M., Murrieta R.A., Aboellail T.A., Campbell C.L. (2019). Bat influenza viruses transmit among bats but are poorly adapted to non-bat species. Nat. Microbiol..

[19] Rasweiler J., Badwaik N.K. (1996). Improved procedures for maintaining and breeding the short-tailed fruit bat (Carollia perspicillata) in a laboratory setting. Lab. Anim..

[20] Rasweiler J.J., Cretekos C.J., Behringer R.R. (2009). The Short-Tailed Fruit Bat Carollia perspicillata: A Model for Studies in Reproduction and Development. Cold Spring Harb. Protoc..

[21] Rasweiler J.J., Cretekos C.J., Behringer R.R. (2009). Feeding Short-Tailed Fruit Bats (Carollia perspicillata). Cold Spring Harb. Protoc..

[22] Grund C., Hoffmann D., Ulrich R., Naguib M., Schinköthe J., Hoffmann B., Harder T., Saenger S., Zscheppang K., Tönnies M. (2018). A novel European H5N8 influenza A virus has increased virulence in ducks but low zoonotic potential. Emerg. Microbes Infect..

[23] Hoffmann B., Depner K., Schirrmeier H., Beer M. (2006). A universal heterologous internal control system for duplex real-time RT-PCR assays used in a detection system for pestiviruses. J. Virol. Methods.

[24] Toussaint J.F., Sailleau C., Breard E., Zientara S., De Clercq K. (2007). Bluetongue virus detection by two real-time rt-qpcrs targeting two different genomic segments. J. Virol. Methods.

[25] Moreira É.A., Locher S., Kolesnikova L., Bolte H., Aydillo T., García-Sastre A., Schwemmle M., Zimmer G. (2016). Synthetically derived bat influenza A-like viruses reveal a cell type- but not species-specific tropism. Proc. Natl. Acad. Sci. USA.

[26] Yewdell J.W., Frank E., Gerhard W. (1981). Expression of influenza A virus internal antigens on the surface of infected P815 cells. J. Immunol..

[27] Brandtzaeg P., Kiyono H., Pabst R., Russell M.W. (2008). Terminology: Nomenclature of mucosa-associated lymphoid tissue. Mucosal Immunol..

[28] Schuh A.J., Amman B.R., Sealy T.K., Spengler J.R., Nichol S.T., Towner J.S. (2017). Egyptian rousette bats maintain long-term protective immunity against Marburg virus infection despite diminished antibody levels. Sci. Rep..

[29] Baker M.L., Schountz T., Wang L.F. (2013). Antiviral immune responses of bats: A review. Zoonoses Public Health.

[30] Schountz T., Baker M., Butler J., Munster V.J. (2017). Immunological Control of Viral Infections in Bats and the Emergence of Viruses Highly Pathogenic to Humans. Front. Immunol..

[31] Campos A.C.A., Góes L.G.B., Moreira-Soto A., De Carvalho C., Ambar G., Sander A.-L., Fischer C., Da Rosa A.R., De Oliveira D.C., Kataoka A.P.G. (2019). Bat Influenza A(HL18NL11) Virus in Fruit Bats, Brazil. Emerg. Infect. Dis..

